# Supplementary material for: Regulation of polyamine interconversion enzymes affects α-Synuclein levels and toxicity in a Drosophila model of Parkinson’s Disease
Source: NPJ Parkinsons Dis. 2025 Aug 6;11:231. doi: 10.1038/s41531-025-01087-9 (PMC12328646; doi:10.1038/s41531-025-01087-9)
Supplement: Supplementary file 1 — Supplementary information [file 41531_2025_1087_MOESM1_ESM.pdf]

Regulation of polyamine interconversion enzymes affects α-Synuclein levels and toxicity in a *Drosophila* model of Parkinson's Disease

Bedri Ranxhi[1], Zoya R. Bangash[1], Zachary M. Chbihi[1], Zaina Qadri[1], Nazin N. Islam[1], Sokol V. Todi[1,2], Peter A. LeWitt*[1,2,3], Wei-Ling Tsou*[1]

1- Department of Pharmacology, Wayne State University School of Medicine

2- Department of Neurology, Wayne State University School of Medicine

3- Department of Neurology, Henry Ford Health Systems, Detroit, Michigan

*Correspondence

Wei-Ling Tsou: wtsou@wayne.edu, ORCiD 0000-0001-9136-2581

Peter A. LeWitt: aa1142@wayne.edu, ORCiD 0000-0002-4976-4445

540 E Canfield, Scott Hall Rm 3108, Detroit, MI 48201, USA.

(Wei-Ling Tsou and Peter A. LeWitt contributed equally to corresponding authorship):

Supplementary information

Supplementary Figure 1:

A

Primers:

ODC1 CG8721
ODC1-F CGTATCATCTTCGCCAATCCCT
ODC1-R GCTCTTGAATCTCACGATCAGGT

SRM CG8327
SRM-F CCACGTGTTGAGCCAATCTTATC
SRM-R GTCCATAGGTTTCGGTTTCAACG

SMS CG4300
SMS-F TTCAAATCATGCACTCCAAGACC
SMS-R ATCACCACATGCTTCGGATTCTC

SMOX CG7737
SMOX-F TATCTAGTCGCCTGAAAGCCATC
SMOX-R GTTGACAAAGAACTCACTGGCC

SAT1 CG4210
SAT1-F TCCATGATTCAAGAACTGGCTGA
SAT1-R CACGTTGAGTATGCCTTGTAGC

PAOX. CG8032
PAOX-F AAGCGATGTGTTTGTACCAGTTG
PAOX-R TAGAAACTGGAATGGGTGTGCTC

ATP13A2 CG32000
ATP13A2-F CAGTCGGTCGATTTTATGCACAA
ATP13A2-R GAAAAGGAAGTGATCGAATGGCA

SLC7A2 CG7255
SLC7A2-F GTTTCTTGGGCAGCTACTTTGAC
SLC7A2-R ACCCACCAGATCCGATAGTAGAA

RP49 CG7939
rp49-F AGATCGTGAAGAAGCGCACCAAG
rp49-R CACCAGGAACTTCTTGAATCCGG

B

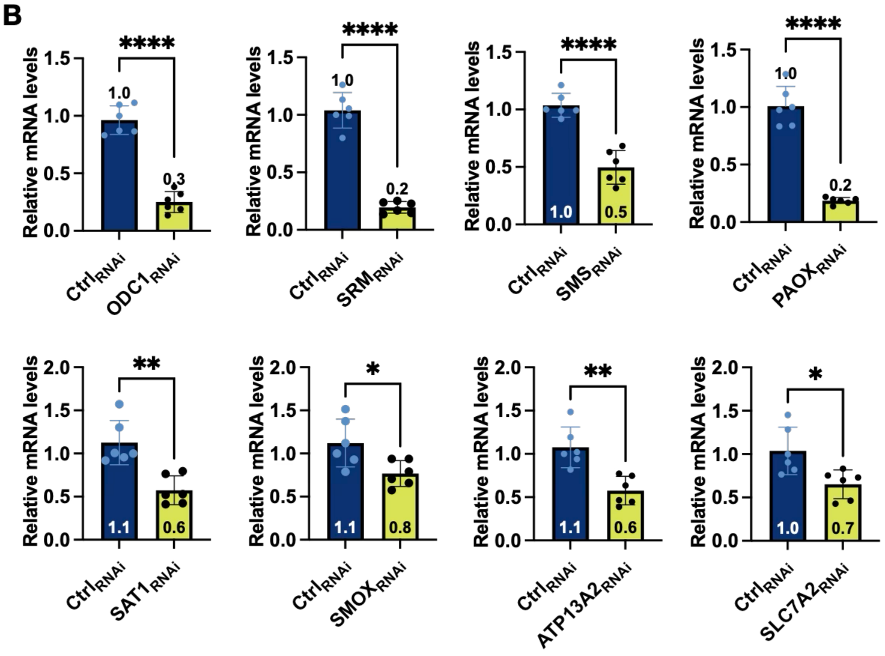

Supplementary Figure 1: mRNA levels measured by qRT-PCR

(A) List of target genes, their corresponding CG numbers in *Drosophila*, and the primer sequences used for qRT-PCR.

(B) Quantification of mRNA levels from qRT-PCR analysis. Mean expression values are displayed on top or within each bar. Total RNA was extracted from 14 fly heads per sample (6 samples per group) using TRIzol reagent (Invitrogen, Waltham, MA, USA) and treated with TURBO DNase (Invitrogen) to remove genomic DNA contamination. cDNA synthesis was performed using the High-Capacity cDNA Reverse Transcription Kit (ABI, Waltham, MA, USA). Quantitative PCR was carried out using the StepOnePlus Real-Time PCR System with Fast SYBR Green Master Mix (ABI). Rp49 served as the internal control. Statistical analysis was performed using an unpaired two-tailed Student's t-test in GraphPad Prism (San Diego, CA, USA). Significance: ns (not significant), * ($p < 0.05$), ** ($p < 0.01$), *** ($p < 0.001$), **** ($p < 0.0001$).

Supplementary Figure 2:

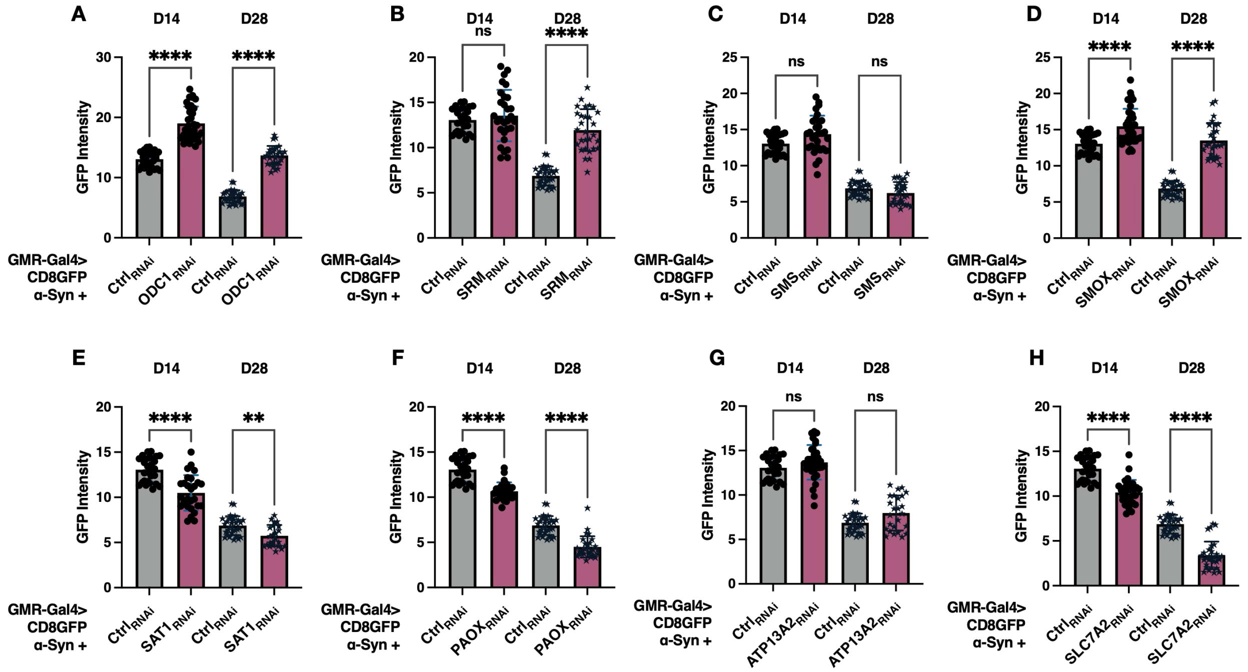

Supplementary Figure 2: Quantification of CD8GFP fluorescence intensity showing that knockdown of polyamine pathway enzymes modulates eye integrity in the male α-Syn *Drosophila* model.

(A-H) Quantification of GFP fluorescence intensity from male fly eye images at days 14 and 28 was performed using ImageJ. Sample size: N ≥ 15 per condition. Statistical analysis was conducted using Brown-Forsythe and Welch ANOVA tests. Significance: ns (not significant), * ($p < 0.05$), ** ($p < 0.01$), *** ($p < 0.001$), **** ($p < 0.0001$).

Supplementary Figure 3:

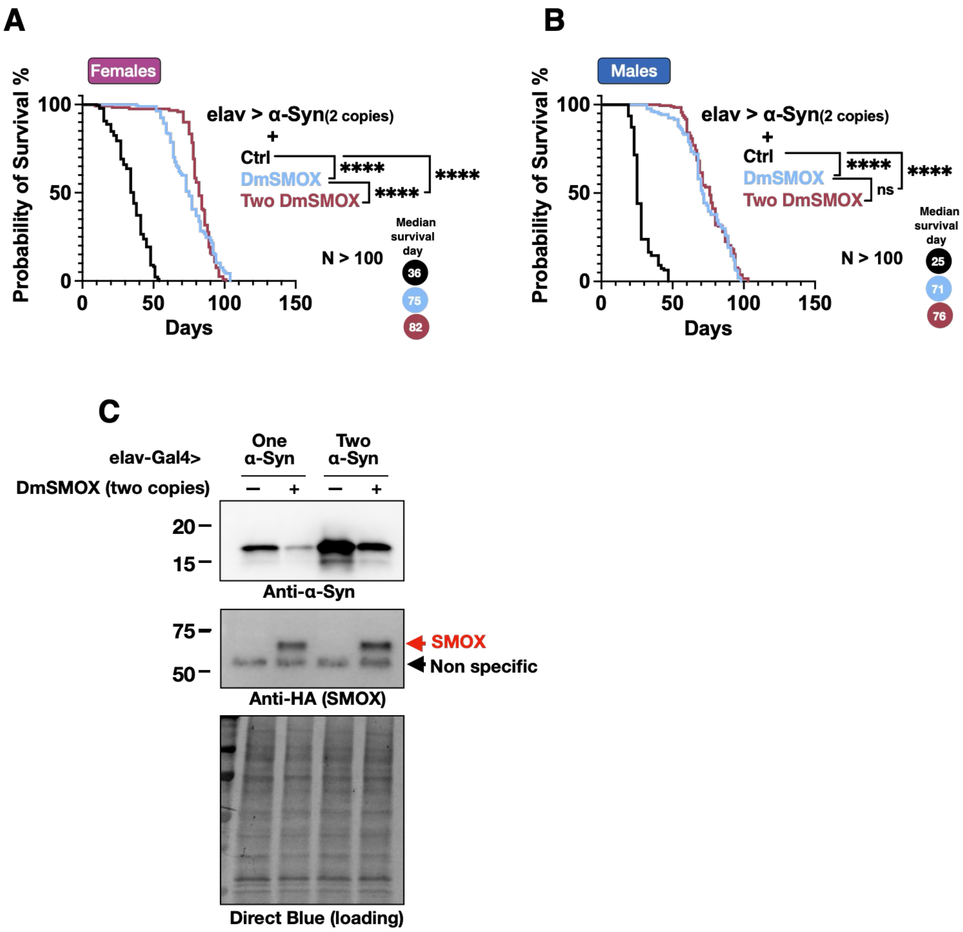

Supplementary Figure 3: Two copies of DmSMOX overexpression improves survival and reduces α-Syn protein levels in *Drosophila*.

(A, B) Survival curves of flies with pan-neuronal expression of two copies of α-Syn and either no DmSMOX (black), one copy of DmSMOX (blue), or two copies of DmSMOX (red) in females **(A)** and males **(B)**. Median survival days are indicated in each group label. Statistical analysis was performed using the Gehan-Breslow-Wilcoxon test. Significance levels: ns (not significant), *p < 0.05, **p < 0.01, ***p < 0.001, ****p < 0.0001. **(C)** Western blot analysis of α-Syn protein levels in flies expressing one or two copies of α-Syn, with or without two copies of DmSMOX. The black arrowhead indicates non-specific bands, while the red arrow marks the SMOX-specific band. Total protein loading is shown in the lower panel.

Uncropped and unprocessed western blots:

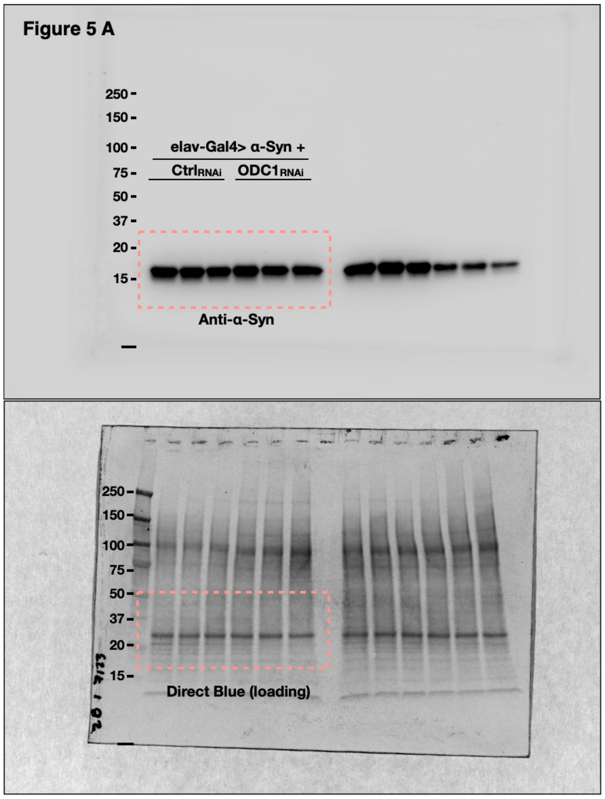

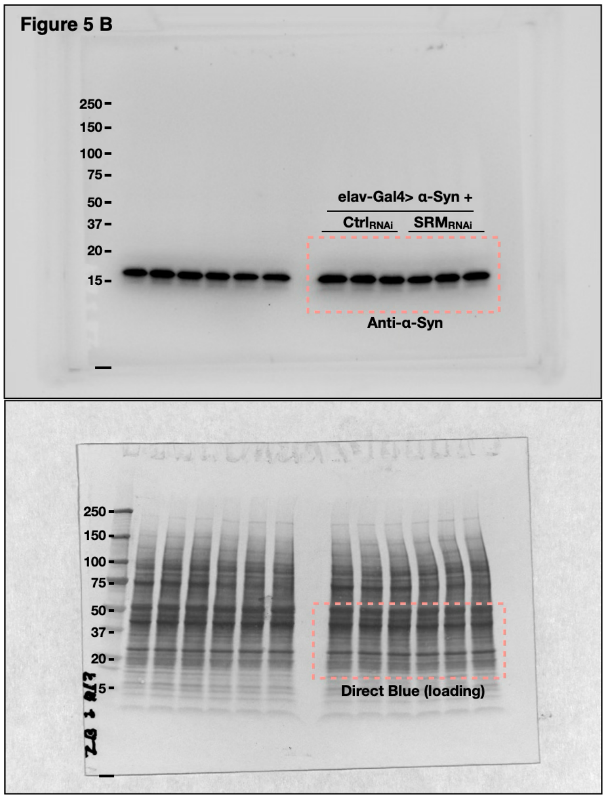

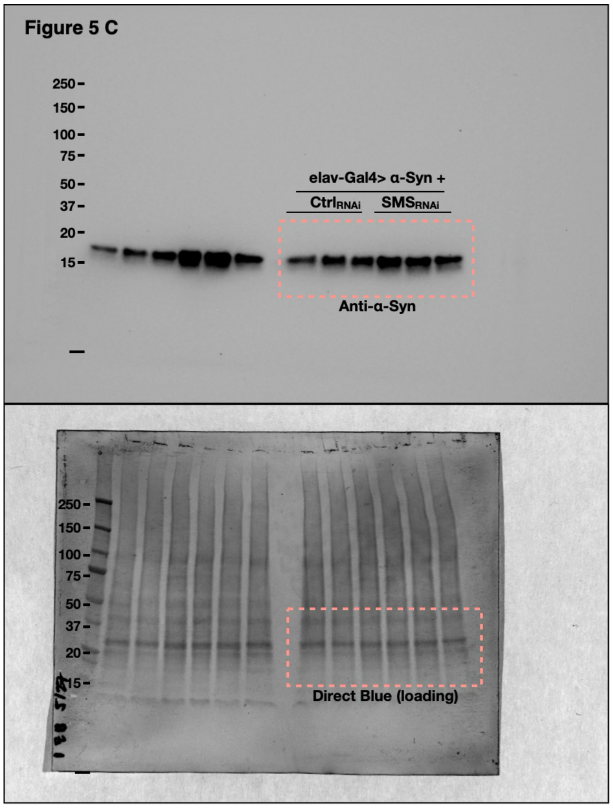

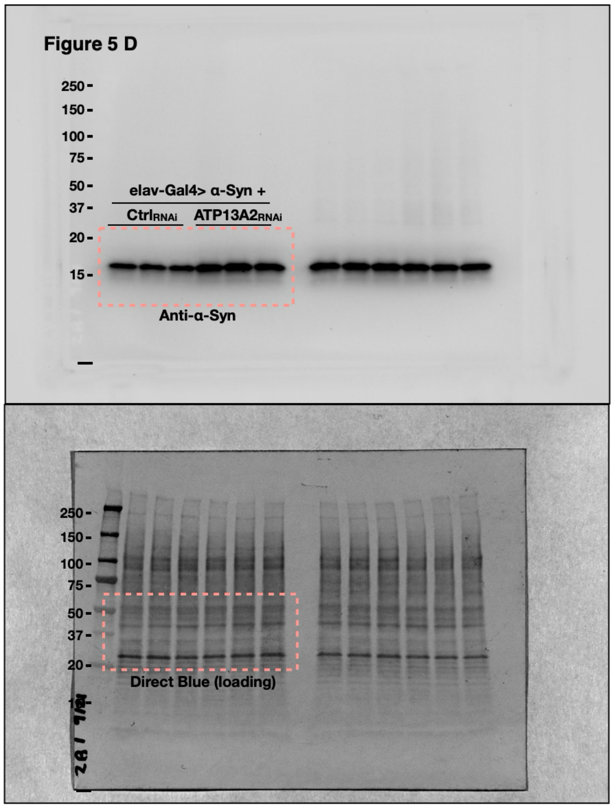

Figure 5 E

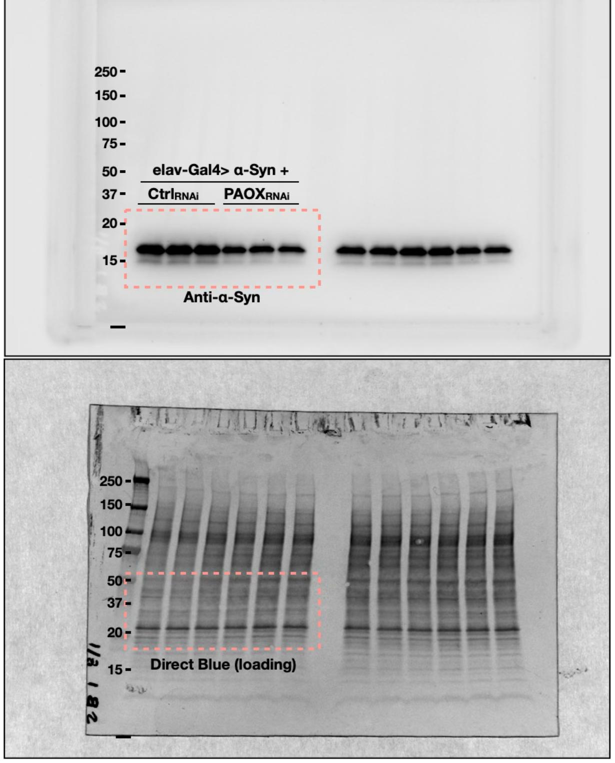

Figure 5 F

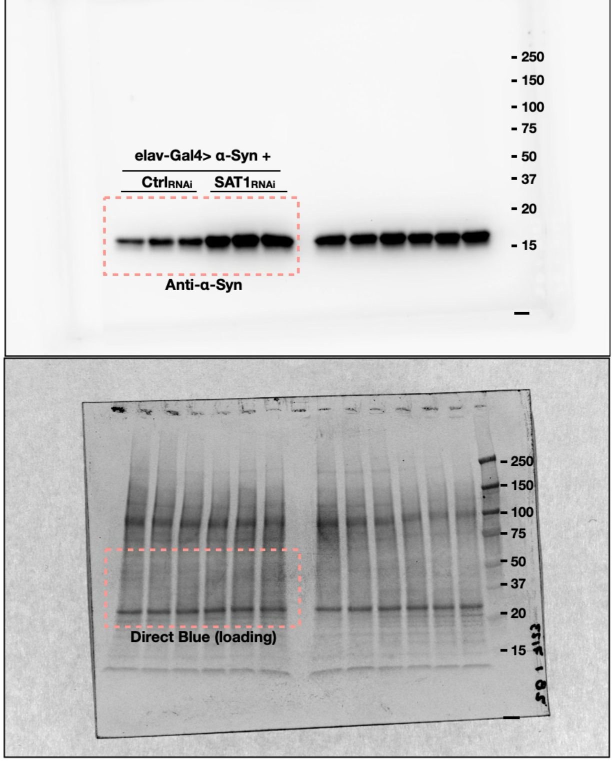

Figure 5 G

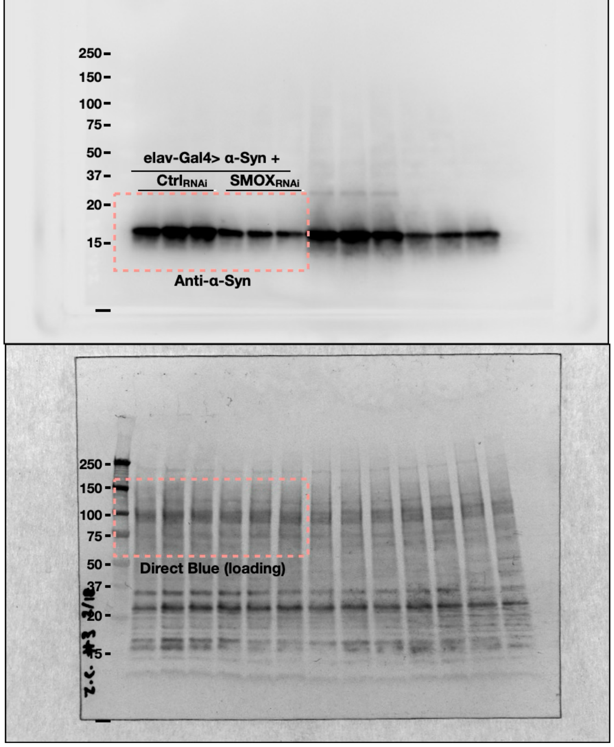

Figure 5 H

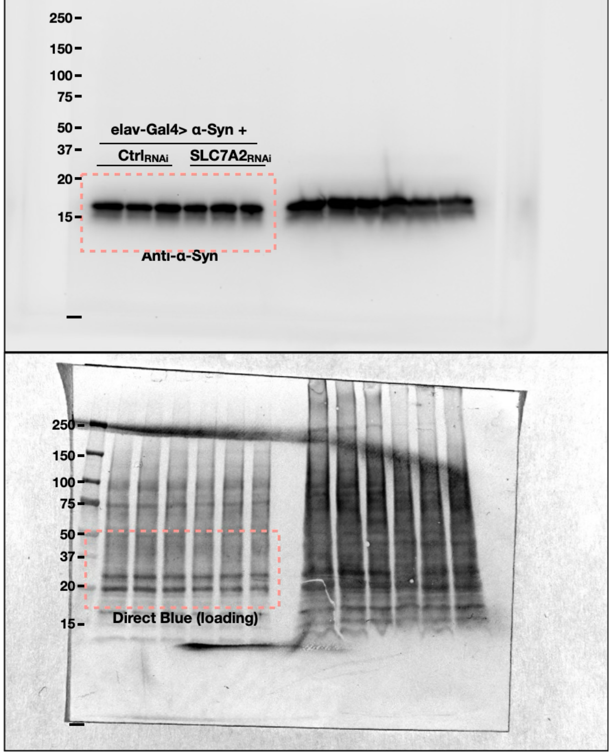

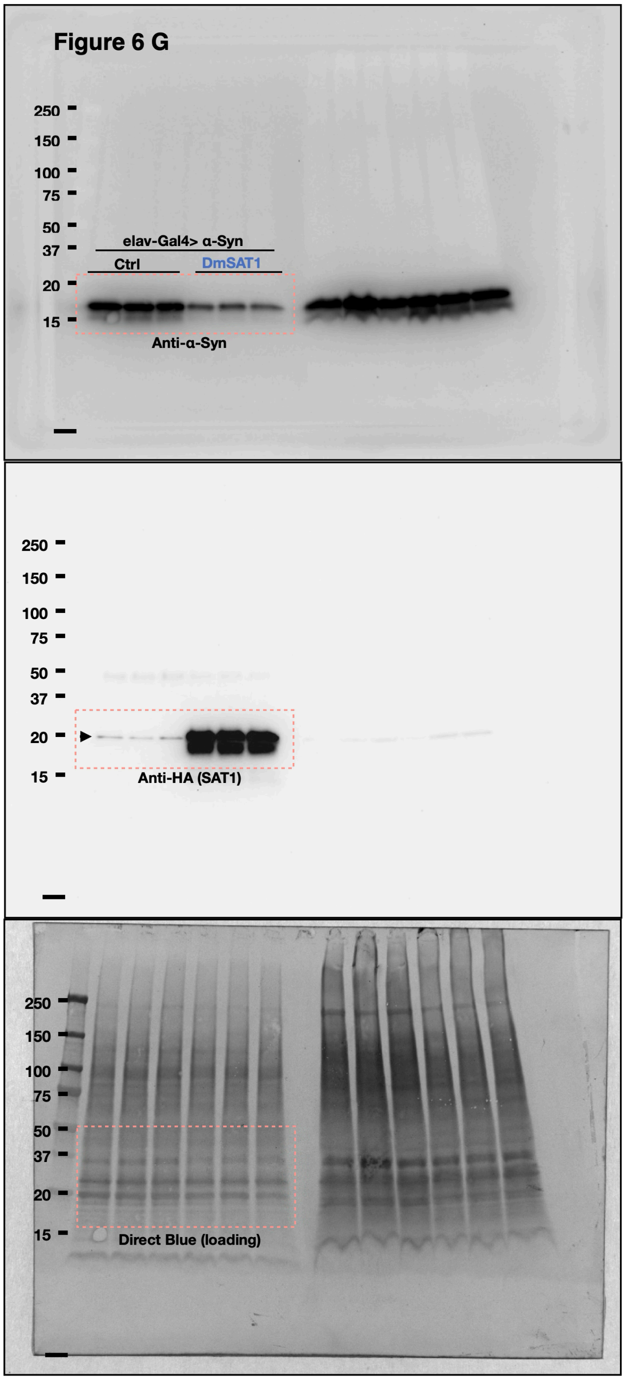
Figure 6 G
250
150
100
75
50
37
20
15
elav-Gal4> α-Syn
Ctrl
DmSAT1
Anti-α-Syn
Anti-HA (SAT1)
Direct Blue (loading)

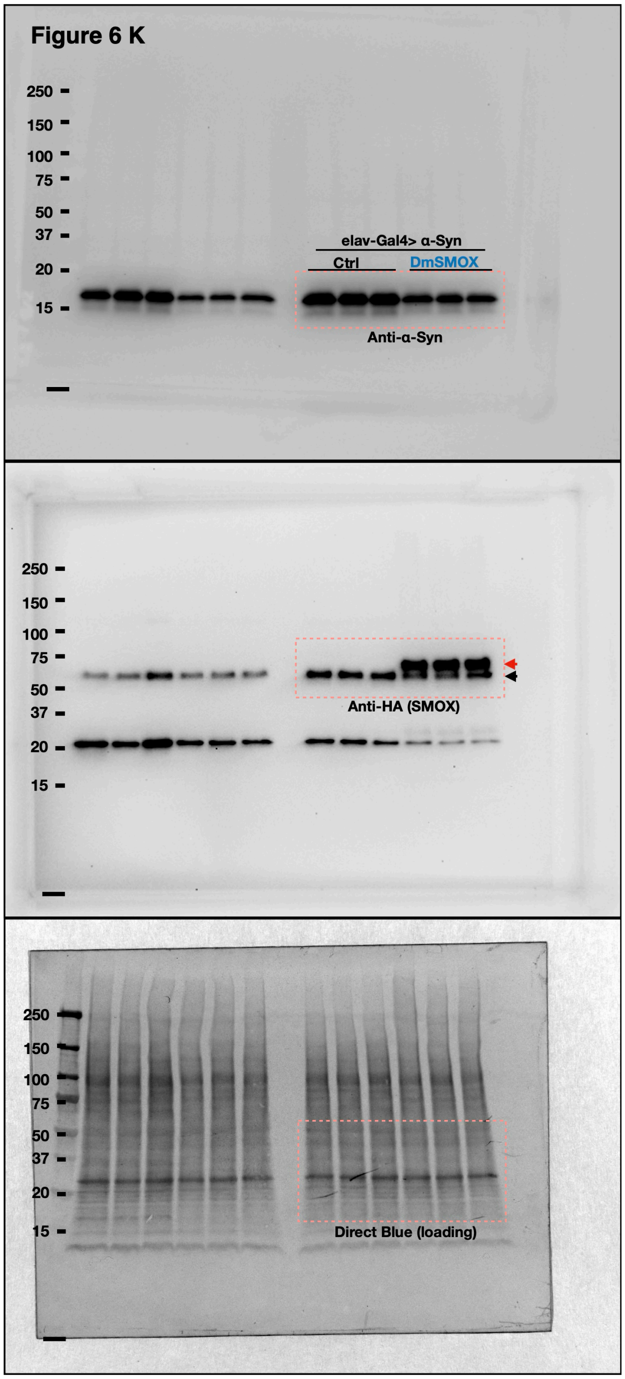
Figure 6 K
250
150
100
75
50
37
20
15
elav-Gal4> α-Syn
Ctrl
DmSMOX
Anti-α-Syn
Anti-HA (SMOX)
Direct Blue (loading)
